# Assessing the Bacterial Communities Composition from Differently Treated Agarwood via 16S rRNA Gene Metabarcoding

**DOI:** 10.3390/life12111697

**Published:** 2022-10-25

**Authors:** Yichen Wang, Mubasher Hussain, Xincheng An, Xiaojun Jiang, Runqian Mao

**Affiliations:** 1 Guangdong Key Laboratory of Animal Conservation and Resource Utilization, Guangdong Public Laboratory of Wild Animal Conservation and Utilization, Guangdong Engineering Research Center for Mineral Oil Pesticides, Institute of Zoology, Guangdong Academy of Sciences, Guangzhou 510260, China; 2 Guangdong Xialiang Health Regimen Agricultural Technology Co., Ltd., Guangzhou 510445, China

**Keywords:** incense formation, medicinal plant, microbiota, plant–microbe interactions

## Abstract

Agarwood (*Aquilaria sinensis*) is one of the most important resin-containing plants used to produce agar around the world and it is a precious herbal medicine usually combined with other herbs. In this study, we used the Illumina sequencing technique to explore the agarwood bacterial community structure from four different incense formations of agarwood, including healthy agarwood, drilling agarwood, liquid fermentation agarwood, and insect attack agarwood. Our results showed that 20 samples of three different incense-formation methods of agarwood and healthy agarwood acquired 1,792,706 high-quality sequences. In-depth investigation showed that when the diversity of agarwood bacterial species was higher, the agarwood incense quality was higher as well. Among healthy agarwood, drilling agarwood, fermentation agarwood, and insect attack agarwood, the bacterial community structure had significant changes. Natural agarwood, such as insect attack agarwood, kept more bacterial community structure, and the incense quality was better. Furthermore, we observed that in the healthy agarwood, *Amnibacterium* and *Delftia* were the predominant bacteria. *Actinoplanes*, *Bordetella*, and *Sphingobacterium* were the dominant bacteria in the drilling agarwood. Additionally, *Pelagibacterium* and *Methylovirgula* were some of the main bacteria in the fermentation liquid agarwood and the insect attack agarwood, while *Cellulomonas* and *Aeromicrobium* were the dominant bacteria. This research provides a basis for further research into the underlying mechanisms of incense production, as well as the bacterial community applications of agarwood production.

## 1. Introduction

**Figure 1:**
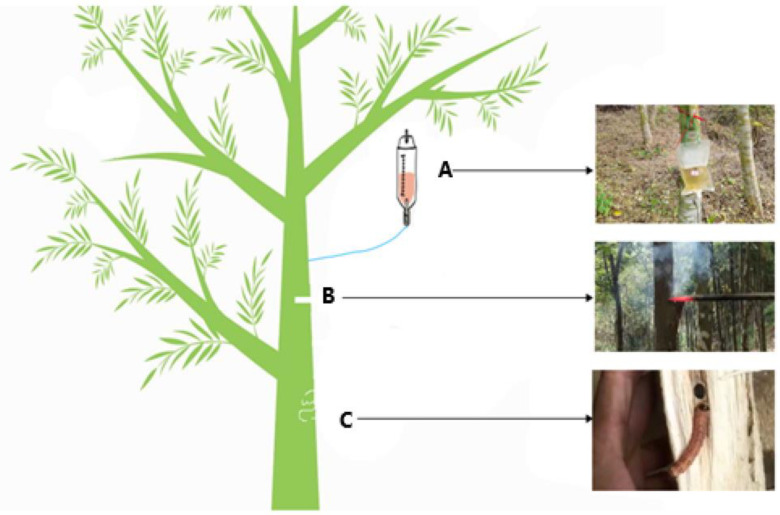
Different methods for agarwood production. (**A**) Fermentation liquid agarwood (FLA): chemical reagents with bacterial fermentation liquid together; (**B**) drilling agarwood (DA): hot iron drilling holes for more than 2 years among the same tree; (**C**) insect attack agarwood (IA): the agarwood from insect attack holes among the same tree for more than 2 years.

*Aquilaria sinensis* is one of the most important resin-containing plants for producing agar (a resin from agarwood) in China, and agarwood is a certified resource listed in traditional herbal medicine [1]. Thus, agarwood is a valuable medicinal material with a broad market demand in many fields. More than 20 million planted *A*. *sinensis* trees are estimated to be distributed in the Guangdong, Yunnan, and Hainan Provinces, and more than a quarter of agarwood have grown for more than 5 years and can produce agar. Its effects may relieve pain, lessen vomiting, and reduce asthma [2]. Agarwood (the trunk from the *Aquilaria sinensis* tree) is traditionally graded according to the senses of the body. It is evaluated by its color resin content, taste, sinkage/density, weight/texture, scent/aroma, sense of oiliness, methods of inducing incense (such as IA/DA/FLA), and formation time. Regarding resin color, agarwood from different species could contain diverse agar colors, such as black, golden, brown, yellow, and red. The agar content and grade are higher when the agarwood trunk is browner or blacker. A healthy agarwood tree cannot produce agar. It is only when the plant is damaged that it is possible to produce agar (a variety of secondary metabolites). Examples of damage include a lightning strike, burning, an insect attack (IA) (Figure 1), or a moth-eating or microbial invasion of natural factors. Insect attack agarwood is formed for a long time under the erosion, injury, and stress of insects, including micro-organisms and other factors, and it is recognized as one of the most high-quality natural agarwood options in the industry [3]. Although the quality of natural agarwood is the best, the speed of agar formation is very slow, which leads people to explore the methods of artificial agarwood formation, such as cutting, burrowing, drilling (DA), fermentation liquid erosion (FLA), and other artificial agarwood-inducing methods that have been developed [4,5]. Revealing the mechanism of incense-formation in wounded trees of agarwood is vital to find an effective stimulation method for *A. sinensis*.

Different methods for an agarwood wounding result in diverse qualities of agar-wood [6]. The bacterial community and plants form a joint relationship between micro-organisms in the long-term coevolution process that widely exists in plant tissues. Studies have revealed that bacteria in plants play a vital role in the formation of agar [7,8,9,10]. In order to simulate the natural state, the agarwood tree is attacked by micro-organisms and forms agar.

High-quality agarwood can be sold for USD 100,000 per kg, while poor-quality agarwood can only be sold for USD 100 per kg. As a high-quality and high-grade agarwood essential oil, it can even be sold for USD 1,500 per 11.7 g. The higher the grade of the agarwood, the richer the aroma of the wood [11,12,13].

Han et al. [14] used the conventional pure culture approach to investigate the variety of soil micro-organisms in the root of Paeonia ostii. The findings revealed that the root bacterial community structure varied significantly among the several tree peony strains (Centaurea cyanus L. and Paeonia ostii T. Hong et J. X. Zhang). Paeonia ostii’s root microbial community was studied by Xue et al. [15] using a polymerase chain reaction-denaturing gradient gel electrophoresis (PCR-DGGE) to determine its composition and its relationships with different tree peony strains and planting dates. The results demonstrate that the soil microbial community structure was influenced by both tree peony strains and planting years, with the latter being higher than the former. The 16S rRNA has been widely used to investigate the microbial levels in different agarwood. The application of a culture-free technique based on 16S rRNA gene analysis for the study of plant bacteria can obtain more comprehensive and objective information of microbial diversity and structure [16,17,18]. Bhore et al. [9] have investigated the microbial levels in different agarwood that have adopted the methods of cloning and PCR. Due to the limitations of this approach, modern techniques are rapid and sensitive, and their method is being improved for microbial species identification. The advent of next-generation technology has enabled the targeting of 16S rRNA hypervariable regions for microbiota parallel, and its full-scale information on bacterial composition can be acquired from different types of agarwood. Characterizing the bacterial community from the different types of the agarwood grounded on the examination of the 16S rRNA gene sequences by means of Illumina sequencing determines the extent of the presence of a xenobiotic degrading gene pool in the community constitution of bacteria across the agarwood [16,17,18].

Density/sinkage: small pieces of agarwood can sink in water, which means the pieces have more agar and thickness. When the traditional method is used to judge the agarwood, we usually place a small piece of an agarwood chip into water, and then the agarwood chip is classified into three simple grades: sinkage, half-sinkage, and floating. The better the quality of the agarwood chip, the lower they sink in water. Resin content: resin content is the main chemical constituent in agarwood (such as chromone, sesquiterpene, saponis etc.) [19]. The trunk of incense-forming agarwood trees has a higher density with black, red, and brown colors than healthy agarwood with its whitish color. Usually, the more resin content there is, the more valuable the agarwood is. We usually estimate agarwood quality according to the resin content on its surface. Scent/aroma: the scent/aroma of the *Aquilaria* tree is pleasing and attractive. Tastes: normally, first-class agarwood tastes sweetened, sour, bitter, or salty. Inferior agarwood tastes like chewing sawdust. Incense-formation methods and incense-formation time: *Aquilaria* trees can produce agar via many incense-formation techniques. For example, when an agarwood tree is wounded by artificial lesions (DA), chemical invasion (FLA), and insect/microbe attack (IA), it will produce different kinds of resin componence. Some people suspect that the incense produced by these different incense-forming techniques is related to the structure of the bacteria. Furthermore, it is generally believed that the older the tree is and the longer it takes to form incense, the more resin-bearing substances can be accumulated. The agarwood quality grades system is shown in Table 1.

**Figure 2:**
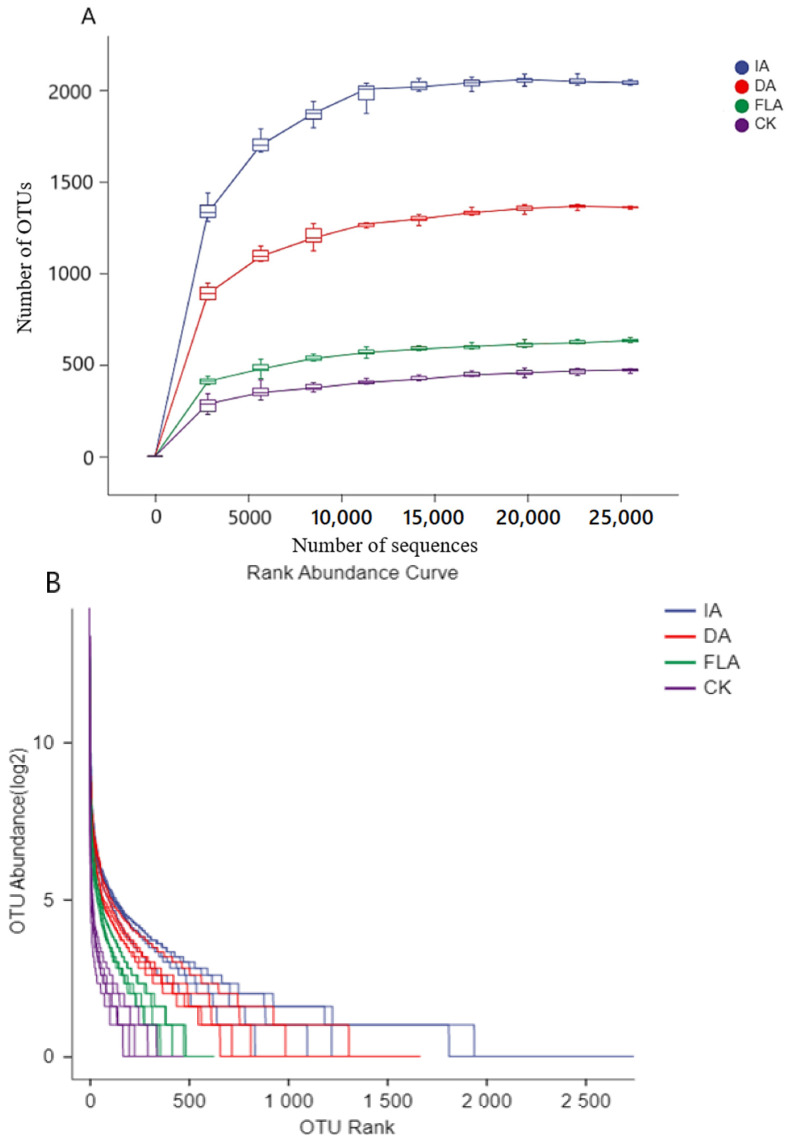
Analyses of species diversity curves of different agarwood, including: (**A**) rarefaction curve and (**B**) rank abundance curve.

There are few reports about the artificial cultivation of agarwood. Most of them focus on drilling, cutting, fermentation liquid dripping, and implanted bacteria, but the natural agarwood is superior among all the agarwood, and the insect attack agarwood is the first-class product in the natural agarwood [20,21]. Based on previous studies, we hypothesized that the agarwood quality is related to the environment, and the key is to reveal the condition changes of the microbial community in the process of incense formation. However, the amount of the genomic information of the microbial community that is available from agarwood is limited. The key to testing this hypothesis is to uncover the mechanism of the bacterial community’s effect on incense formation in agarwood. In this study, we used Illumina sequencing in order to (1) provide 16S rRNA detailed insight into the different induction methods of agarwood and healthy agarwood (Figure 1); (2) find the relationship between the quality of agarwood and the bacterial community; and (3) identify micro-organisms that may be beneficial to incense formation.

## 2. Materials and Methods

### 2.1. Sample Collection

We used a 6-year-old *Aquilaria sinensis* (NCBI genomes taxonomy ID:210372) tree grown in Maoming’s plantation garden in China’s Guangdong Province. We then collected a variety of agarwood created via the different types of incense formation. These include the following. Insect attack agarwood (IA): the agarwood from insect attack holes among the same tree for more than 2 years; drilling agarwood (DA): the hot iron drilling holes among the same tree for more than 2 years; fermentation liquid agarwood (FLA): the chemical reagents with bacterial fermentation liquid combined together; and control treatment (CK): the healthy agarwood. Two years later, when agar formed, agarwood samples were collected by cutting off the wood from the agarwood trunk. The cutting site was about 0.5–1 m above the ground. We scraped the agarwood around where the agar formed. Each sample was 1 g, with five repeated samples, and a total of 20 samples. The different incense-formation trees of *A*. *sinensis* were collected, including the insect attack agarwood (IA), the drilling agarwood (DA), the fermentation liquid agarwood (FLA), and the control treatment (CK) or the healthy agarwood. 

### 2.2. Amplification Agarwood Microbiome Analysis

The genomic DNA (gDNA) of the sample was extracted via the Cetyltrimethylammonium Bromide (CTAB) method [20]. The bacterial 16S rRNA V4 region primers (515F: 5′-GTGYCAGCMGCCGCGGTAA-3’ and 806R:5’-GGACTACNVGGGTWTCTAA-3’) were used to amplify and purify the PCR products. Amplification was performed under the following conditions: initial denaturation at 95 °C for 3 min, 30 cycles at 95 °C for 30 s, then 52 °C for 30 s, 72 °C for 45 s, and a final extension at 72 °C for 5 min. The glue recovery equipment was provided by the QIAGEN Company, and it was used to recover the product for the target strip. The PCRs were carried out using the Phusion^®^ High-Fidelity PCR Master Mix (New England Biolabs, Ipswich, MA, USA). The PCR products were mixed in equidensity ratios and purified.

### 2.3. Library Construction and HiSeq Sequencing

The library was constructed with the Agilent High-Sensitivity DNA Kit library construction kit, and the constructed library was calculated with the Quant-iT PicoGreen dsDNA Assay Kit and the Promega QuantiFluor. After the library was established, the Illumina sequencer was used for the processor sequencing [22].

### 2.4. Statistical Analysis and Data Records

The read quality was preprocessed with FLASH [23], and the splicing sequence was the raw tags data. According to 97% similarity, operational taxonomic units (OTUs) of both effective tags among all the samples were carried out using the Uparse [24] software. The Mothur method [25] and the SSUrRNA base date [26] of SIL VA were used for species, and the annotation analysis and the comparison threshold of this paper were set to 8~1. The taxonomic data were obtained, and the bacterial community composition of each sample was calculated at all classification levels. We obtained the phylogenetic associations of all OTUs typical sequences via fast multi-sequence alignment using MUSCLE [15,27] software. Finally, the information of the sample was homogenized. A species diversity curve analysis was used to analyze the species richness and the evenness of the agarwood samples. The statistics of the number of common and unique species in the different methods of agarwood were visualized with a Venn diagram. Alpha diversity (including Chao1 index, Observed species, Shannon index, and Simpson index) was used to analyze the richness of the bacterial communities in the agarwood samples. A principal coordinate analysis (PCoA) was used to compare and analyze the repeatability of the bacterial community composition in the different agarwood samples. Linear discriminant analysis effect size (LEfSe) analysis was used to identify the characteristics of the different types of agarwood bacteria and the associated categories and look for biomarkers with statistical differences between the groups. Diversity analyses and the difference test were conducted to determine the composition of the bacterial community in the sample.

## 3. Results

### 3.1. Species Diversity Curve Analysis

Species diversity curve analyses (including the rarefaction curve and the rank abundance curve) were used to analyze the agarwood sample’s species richness and evenness (Figure 2). From the dilution curve, the density distribution law of the bacterial diversity is as follows: insect attack agarwood > drilling agarwood > fermentation liquid agarwood > control (Figure 2A), which shows that the bacterial diversity of the insect attack agarwood is higher than the artificial agarwood (DA and FLA).

The results of the rank abundance curve (Figure 2B) showed that the vertical curve of the agarwood prepared by the healthy trunk and the fermentation liquid was smoother than the other two groups of the agarwood formation samples (IA and DA), which meant that the change in the bacterial abundance was more uniform than IA and DA. This result also showed that the bacterial abundance will greatly reduce in a healthy trunk or when the trunk is not exposed to the air. Moreover, it produced the incense without microbial damage, so we speculate that the micro-organisms are not a necessary condition for agarwood incense formation.

The results showed that the bacterial species diversity of the healthy agarwood was the lowest compared to the other incense-formation agarwood (IA, DA, and FLA), and the bacterial diversity of the natural insect attack agarwood was higher than the other artificial agarwood (DA and FLA).

### 3.2. Venn Graph Analysis

**Figure 3:**
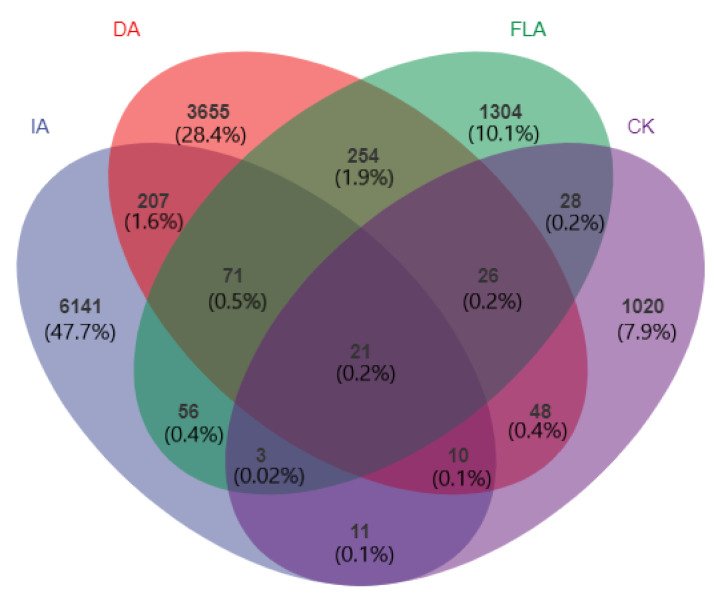
Venn graph analysis of different types of agarwood based on operational taxonomic units.

The number of common and unique bacterial species in all the samples with the different incense-formation methods and the healthy agarwood (CK) was calculated via the Venn graph method (Figure 3). The results showed 21 species of OTUs in all the agarwood samples, accounting for 0.2% of the total samples. The OTUs that were unique to the insect attack agarwood (IA) were the highest, accounting for 6141 (47.7% of the total sample OTUs), and the total OTUs of the incense formed by the incense agarwood (IA, DA, and FLA) was 71 (0.5% of the total sample OTUs), and the proportion was 0.5%. Among them, the sample with the healthy agarwood (CK) contained 1020 unique species (7.9% of the total sample OTUs). It can be seen from the results that natural insects attack agarwood (IA) and artificial agarwood (DA and FLA), and that the additional kinds of bacteria in the agarwood trunk were beneficial to the formation of the high-quality incense. The bacteria in the agarwood were not all the same, and cooperation between different bacteria also produced agar, but the quality of the incense was different.

### 3.3. Alpha Diversity Index Analysis Observed Species Index

**Figure 4:**
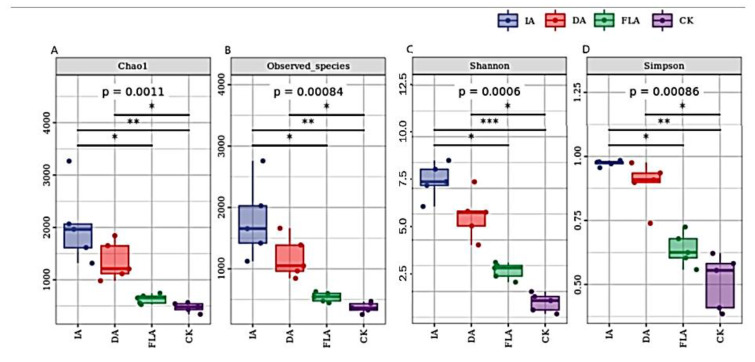
(**A**–**D**) is the analysis of alpha diversity index. * There is significant difference between the two groups (*p* < 0.05); ** *p* < 0.01; *** *p* < 0.001.

Alpha diversity (including Chao1 index, Observed species, Shannon index, and Simpson index) was used to analyze the richness of the bacterial communities in the agarwood samples with the different incense-formation methods (Figure 4). From Chao1 index and Observed species (Figure 4A,B), the results showed that the bacterial species richness of the wild insect attack agarwood (IA) was significantly higher than the artificial agarwood (DA and FLA) and the healthy agarwood (CK) (*p* < 0.01). Shannon and Simpson’s indexes were used to analyze the equality of the bacterial species distribution in the agarwood samples (Figure 4C,D). The results showed that the bacterial species distribution of IA had higher species diversity, and the bacterial species distribution of different treatment groups was significantly different (*p* < 0.01).

### 3.4. Analysis of Bacterial Community Composition and Species Richness

To further analyze the composition of the bacterial species in the samples at the genus level, a heat map analysis of the bacterial community with high abundance in the samples was carried out. (Figure S1 deposited in the supplemental document. The darker the green, the higher the richness, and the darker the brown color, the lower the richness.) The result showed dissimilarities in the bacterial community abundance among the different treatment groups, but there was little difference among the same treatment groups. We can infer from the figure that the bacterial abundance of the IA samples was the highest. There were no fixed dominant bacteria in each repetitive sample, but all the five repeated samples contained *Cellulomonas*, *Aeromicrobium*, *Solirubrobacter,* and *Sphingobium*, etc. (Figure S1 deposited in the supplemental document). The relative abundance analysis of the subordinate relative abundance showed that the abundance of *Halomonas, Aliihoeflea*, *Nesterenkonia*, *Pelagibacterium*, *Methylovirgula*, etc., was higher in the fermentation liquid agarwood materials (FLA). *Microbacterium*, *Actinoplanes*, *Bordetella*, *Sphingobacterium*, *Pseudosphingobacterium,* and *Sphingomonas,* etc., was the highest in the drilling agarwood (DA). The abundance of the bacteria in the healthy agarwood (CK) was lower, and *Amnibacterium*, *Delftia,* and others were the dominant strain (Figure S1 deposited in the supplemental document).

### 3.5. Principal Component Analysis

**Figure 5:**
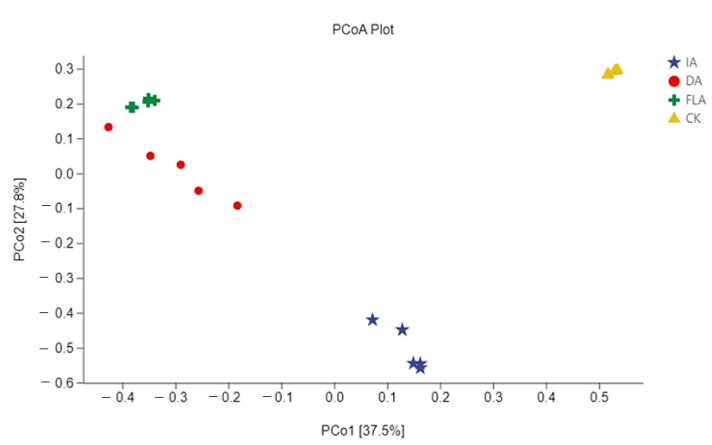
Bacterial community composition in different agarwood samples using principal component analysis.

Principal component analysis (PCoA) was used to compare and analyze the repeatability of the bacterial community composition in the different agarwood samples (Figure 5). The sample point distance of the same treatment group showed the repeatability of the same treatment sample, while the distance of the different groups of samples reflected the discrepancy of the sample distance between the different groups. The results showed that the repeatability of the same treatment sample points was gathered together, which meant that in the same group, the repeatability was strong. It also meant that there were more diversities between the different treatment groups of the samples.

### 3.6. Quality Analysis of Sequencing

A total of 1,850,565 valid sequences were detected in all the samples of the agarwood, of which there were 1,792,706 high-quality sequences, which accounted for 96.68% of the whole sequence (Table S1 deposited in the supplemental document).

We clustered all the high-quality sequences by the Operational Taxonomic Unit (OTUs) and set the parameter to 95% consistency. We then carried out species annotations on the representative sequence of OTUs. All the samples shared 12,855 different OTUs (IA + DA + FLA + CK), with an average of 643 OTUs per sample (Table S1 deposited in the supplemental document).

### 3.7. LefSe Analysis

**Figure 6:**
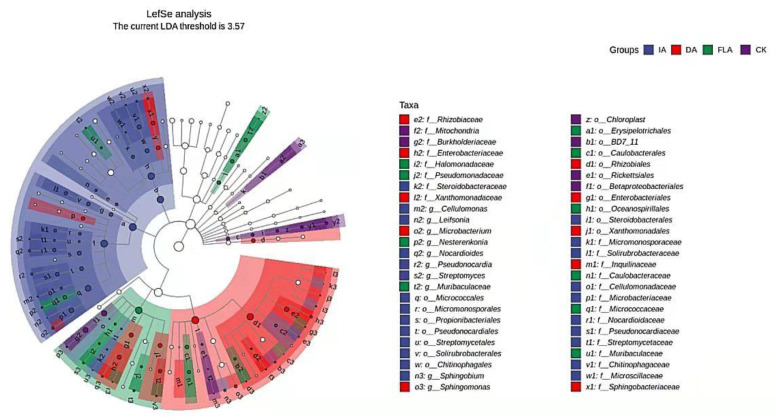
Cladogram by LEfSe analysis.

A linear discriminant analysis effect size (LEfSe) analysis was used to identify the characteristics of the abundance of the different types of agarwood bacteria and the associated categories, looking for biomarkers with statistical differences between the groups. The results showed that *Pseudonocardiaceae* and *Streptomyces* were the main strains in the insect attack agarwood (IA), and *Rhizobiaceae* and *Microbacterium* were the main strains in the drilling agarwood (DA). Further, *Caulobacteraceae* and *Nesterenkonia* were the main strains in the fermentation liquid agarwood (FLA). Finally, *Burkholderiaceae* and *Oxyphotobacteria* were the main strains in the healthy agarwood (CK) (Figure 6).

## 4. Discussion

The quality analysis of sequencing showed that after screening out 1,850,565 functional sequences from all the samples, 1,792,706 high-quality sequences were obtained (Table S1 deposited in the supplemental document).

In the analysis of the species diversity curve (Figure 1), it can be seen that agarwood, mountain agarwood, and others were similar to other fragrant plants [28]. Incense-formation agarwood had higher bacterial diversity than unscented wood, but the change in the bacterial abundance of the healthy, unscented wood was more unified. Therefore, it is speculated that the resin formation of the fragrant plants is closely related to the bacterial abundance, and bacterial species with fragrant materials are more abundant.

The statistical data of the Venn graph analysis (Figure 2) indicated that different types of agarwood had a certain number of common species, but the number of unique bacteria was relatively large. Similar results were obtained by Zhang et al. [22], who observed that the diversity of the bacteria in the healthy agarwood (CK) group was the lowest among all the types of agarwood. The analysis also suggests that to produce higher-quality incense, it is best to have more bacterial communities.

According to the analysis of the Alpha diversity index (Figure 3), the species distribution uniformity and the species diversity of the healthy agarwood (CK) were low, as observed by Chen et al. [29]. At the same time, the other types of incense samples contained a larger flora structure, which also provides a fresh clue for the identification of the artificial agarwood (DA and FLA) and the natural agarwood (IA).

According to the results of the bacterial community composition and the species richness analysis (Figure S1 deposited in the supplemental document), *Acremonium* was the main bacteria observed in the healthy agarwood (CK), and *Penicillium* was the main bacteria in incense agarwood. This result contrasted with the study by Du et al. [30], who observed that *Fusarium* was the dominant bacteria in the stimulating agarwood incense formation. However, our results were similar to those of Huang et al. [10], who found that the dominant bacteria in the healthy agarwood (CK) was *Prevotella*. The dominant bacteria in the incense-formation samples were *Bordetella Clostridium*, *Delftia*, *Altererythrobacter*, *Parabacteroides,* and others. The results showed that the bacterial community of the agarwood was not necessarily the same, whether they were fragrant or healthy. Therefore, our study speculates that these strains could work together to help the agarwood form the incense.

A principal component analysis showed that there were more diversities among different groups (Figure 5), which could be related to the degree and the level of the incense formation (including rot layer, decay and incense transition layer, incense-formation layer, transition layer, and white wood layer), which is also similar to that of mountain-agarwood plants [27,28]. This result also suggests that the incense formation may be completed under the influence of the different bacteria present at the different stages of the agarwood.

The LEfSe analysis (Figure 6) indicated that most of the bacteria enriched in the healthy agarwood (CK) were nonpathogenic bacteria, which was similar to the results obtained by Zhang et al. [22]. On the other hand, most of the bacteria enriched in the incense agarwood were harmful bacteria, which could have the ability to stimulate plants and produce bioactive substances.

There are rich species of bacteria in wood, which can stimulate the growth and the development of plants and resist the invasion of diseases and insect pests [31]. Therefore, it is of great importance to study the bacterial community as a biocontrol bacterium to promote the growth of plants [32]. After the incense was created, the community structure of the bacteria changed obviously, and the change in the bacteria could be involved in the formation of incense. Furthermore, agar-formation wood is rich in secondary metabolites, and it has good antibacterial activity, suggesting that the formation of the incense is closely related to plant immunity. This lays a solid theoretical foundation for exploring the mechanism of incense formation in the later stage [33,34]. Therefore, in this study, the 16S rRNA method was used to explore the community structure and the changes in incense-forming bacteria in different methods and to provide a scientific basis for the rational use of bacteria as biocontrol bacteria and incense-promoting resource bacteria.

## 5. Conclusions

In this study, comprehensive 16S rRNA analyses were performed on healthy agarwood and other types of wounded agarwood. The abundance of the bacterial community and the quality of the agarwood are directly correlated because the higher the abundance, the better the quality. This finding also serves as a means of differentiating between artificial and natural agarwood (IA, DA, and FLA). Therefore, the likelihood of the bacterial community being natural increases with its diversity. Because the resin generated by agarwood after being attacked has an antibacterial function, it is important to note that the bacteria that might cause it to bear incense are not always the same. These findings help to elucidate the molecular mechanism of incense formation and provide us with a better understanding of the bacterial community and the incense formation in agarwood.

## Figures and Tables

**Table 1:** Agarwood quality grades system.

Morphological Characteristic	Grade		
	I (IA)	II (DA)	III (FLA)
Resin color	Black, brown, or red	Yellowish or reddish-brown	Khaki, yellowish, or a little brown
Density/Sinkage g/cm^3^	Dense and solid and sinks in the water ρ ≥ 1	Dense and sinks in the water, but not to the bottom 0.520 ≤ ρ < 1.00	Light and floats on the water 0.380 ≤ ρ < 0.520
Resin content	High	Middle	Little
Room temperature: Scent/Aroma Heating:	Natural fragrance, cool feeling, and frankincense aroma	Slightly obvious fragrance	Weak fragrance
	Strong penetration of the fragrance	Obvious fragrance	Slightly obvious fragrance
Texture/Weight	Hard texture, heavy feeling	The texture is a little hard and a little heavy	Loose texture, sense of lightness
Taste	Bitter, spicy, hemp, sweet, easy to stick to the teeth, no sense of fiber	Sense of fiber and sawdust after chewing	Sawdust after chewing
Incense-formation time	More than 5 years	More than 3 years	More than 1 year

## Data Availability

Not applicable.

## Supplementary Materials

The following supporting information can be downloaded at: https://www.mdpi.com/article/10.3390/life12111697/s1, Figure S1: Analysis of bacterial community composition and species richness in a different type of agarwood at genus level; Table S1: Number of sequences analyzed for 16S rRNA libraries of green genes database.
