# Supplementary material for: Assessing the Bacterial Communities Composition from Differently Treated Agarwood via 16S rRNA Gene Metabarcoding
Source: Life (Basel). 2022 Oct 25;12(11):1697. doi: 10.3390/life12111697 (PMC9693337; doi:10.3390/life12111697)
Supplement: Supplementary file 1 [file life-12-01697-s001.zip › life-1912585-supplementary.pdf]

Table S1. Number of sequences analyzed for 16S rRNA libraries of green genes database.

Type	Sample ID	Fragrant or not	Input	Filtered	Proportion%
Insect attack agarwood	IA1	Yes	76665	76474	99%75
	IA2	Yes	56761	56602	99%72
	IA3	Yes	82439	82159	99%66
	IA4	Yes	99096	98503	99%40
	IA5	Yes	85489	85207	99%67
Drilling agarwood	DA1	Yes	79579	79366	99%73
	DA2	Yes	76851	76270	99%24
	DA3	Yes	74743	74561	99%76
	DA4	Yes	99488	99225	99%74
	DA5	Yes	85564	85327	99%72
Fermentation liquid agarwood	FLA1	Yes	93656	93386	99%71
	FLA2	Yes	10467	10413	99%48
	FLA3	Yes	84168	83704	99%45
	FLA4	Yes	78113	77947	99%79
	FLA5	Yes	91682	91466	99%76
Healthy agarwood	CK1	No	133941	123582	92%27
	CK2	No	144283	132517	91%85
	CK3	No	129660	119670	92%30
	CK4	No	141112	130229	92%29
	CK5	No	126808	116098	91%55
Total			1850565	1792706	96%68

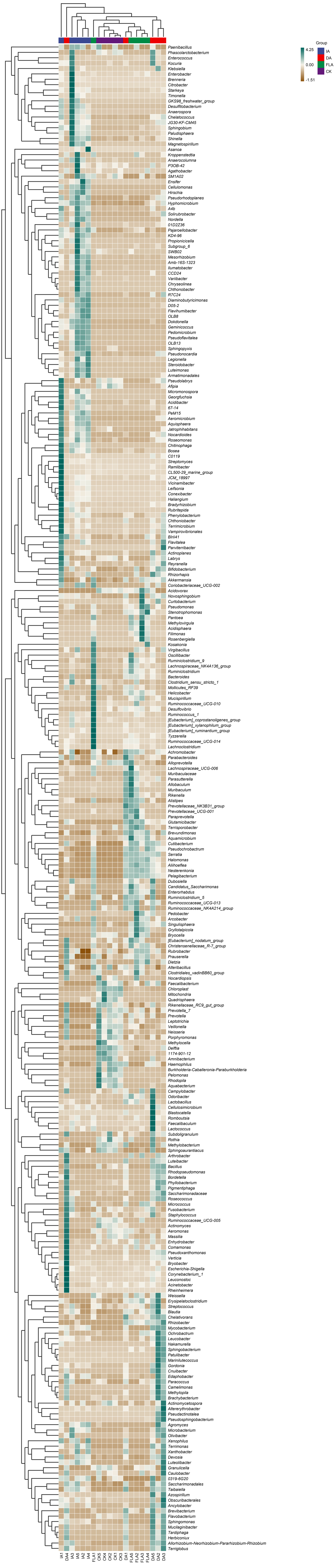

Figure S1. Analysis of bacterial community composition and species richness in a different type of agarwood at genus level
